# Measuring and using information gained by observing diffraction data

**DOI:** 10.1107/S2059798320001588

**Published:** 2020-02-25

**Authors:** Randy J. Read, Robert D. Oeffner, Airlie J. McCoy

**Affiliations:** a Cambridge Institute for Medical Research, Department of Haematology, University of Cambridge, The Keith Peters Building, Hills Road, Cambridge CB2 0XY, England

**Keywords:** information gain, anisotropy, translational noncrystallographic symmetry, diffraction intensities

## Abstract

The information gained by making a measurement, termed the Kullback–Leibler divergence, assesses how much more precisely the true quantity is known after the measurement was made (the posterior probability distribution) than before (the prior probability distribution). It provides an upper bound for the contribution that an observation can make to the total likelihood score in likelihood-based crystallographic algorithms. This makes information gain a natural criterion for deciding which data can legitimately be omitted from likelihood calculations. Many existing methods use an approximation for the effects of measurement error that breaks down for very weak and poorly measured data. For such methods a different (higher) information threshold is appropriate compared with methods that account well for even large measurement errors. Concerns are raised about a current trend to deposit data that have been corrected for anisotropy, sharpened and pruned without including the original unaltered measurements. If not checked, this trend will have serious consequences for the reuse of deposited data by those who hope to repeat calculations using improved new methods.

## Abstract 2

The information content gained by making a diffraction-intensity measurement is a natural criterion for deciding which data make a useful contribution and which can legitimately be omitted from a calculation.

## 1. Introduction   

Likelihood-based methods are now used throughout crystallography to provide a probabilistic treatment of the effects of all sources of error in tasks such as phasing with a model (Read, 1986*a* ▸), experimental phasing (de La Fortelle & Bricogne, 1997 ▸; McCoy *et al.*, 2004 ▸), model refinement (Pannu & Read, 1996 ▸; Bricogne & Irwin, 1996 ▸; Murshudov *et al.*, 2011 ▸) and molecular replacement (McCoy *et al.*, 2007 ▸; Read & McCoy, 2016 ▸). In all of these areas, the introduction of likelihood has led to more powerful and robust methods.

Information gain, described in terms of the Kullback–Leibler divergence or KL-divergence (Kullback & Leibler, 1951 ▸), is a related statistical concept that measures how much is learned when an imperfect measurement is made. This concept has recently become particularly prominent in the context of various applications within machine learning (Bishop, 2006 ▸; Goodfellow *et al.*, 2016 ▸). In crystallography it has only been used rarely, with one example being to evaluate how much different sources of phase information contribute to combined phases (Read, 1986*b* ▸, 1997 ▸).

As discussed in our previous work on this subject (Jamshidiha *et al.*, 2019 ▸), the information content gained by measuring a data set corresponds to the likelihood score that could be achieved with a perfect model, providing an upper limit to what can be achieved with the data in a likelihood-based method. Considered one observation at a time, information provides a way to assess how much value each measurement adds, which is especially relevant for data in which some observations are systematically weakened by effects such as anisotropic diffraction or translational non­crystallographic symmetry (tNCS). This is particularly timely, as there is now a better appreciation that weak data have value, at least up to a point (Karplus & Diederichs, 2012 ▸).

The implementation of our earlier work on information gain for diffraction data was limited to an expected value, *i.e.* what information gain would be expected for a reflection with a particular standard deviation, averaged over all possible intensity measurements that could be made consistent with that size of measurement error. The advantage of this approach is that it lends itself to simple rules: a threshold for useful information gain can be translated into a single number: the corresponding standard deviation of a normalized intensity. A table of normalized standard deviations corresponding to different thresholds of expected information gain was evaluated by numerical integration in the symbolic mathematics program *Mathematica* (Wolfram Research, Champaign, Illinois, USA) and was then used to define thresholds in *Phaser* (McCoy *et al.*, 2007 ▸) without new functions having to be implemented. The disadvantage of this approach is that it neglects the influence of the observed value of the intensity. Here, we explore a more exact calculation in which the actual information gained with each intensity observation is evaluated considering both the intensity and its standard deviation. This allows a true reflection-by-reflection evaluation of the sensitivity of likelihood calculations to an individual observation.

## 2. Computing information gained in measuring diffraction data   

### 2.1. Derivation of information calculation   

The derivation of equations defining per-reflection information gain builds on intermediate steps in our previous work (Jamshidiha *et al.*, 2019 ▸), some results of which are reproduced here for convenience. The equations below are expressed in terms of the normalized intensity *Z* (= *E* ^2^). Note that the expected intensity value used to normalize the intensities should account for overall anisotropy and/or tNCS, if these effects are present.

Information is gained in an experiment when some quantity is known more precisely after carrying out the experiment (measured by the posterior probability distribution for its true value) than before the experiment (measured by its prior probability distribution). As discussed above, this information gain can be evaluated by the KL-divergence. For diffraction data, as discussed previously (Jamshidiha *et al.*, 2019 ▸), it turns out to be more convenient to use a rearrangement based on Bayes’ theorem (1) to express the KL-divergence, *D*_KL_, in terms of the probabilities of the observations rather than the true values, as shown in (2),
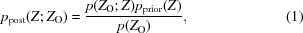


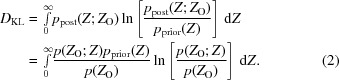



Note from (2) that if the measurement does not alter the prior probability so that the posterior probability is identical (for instance when the standard deviation of the measurement approaches infinity), the logarithm evaluates to zero for all values of the intensity; as expected, no information has been gained. Information gain is expressed in units of nats if the natural logarithm is used, or in bits using logarithm base 2, corresponding to dividing nats by ln(2). In the following, we will use the traditional units of bits for information content on its own. Likelihood is traditionally computed with the natural logarithm, but we will convert it to units of bits when comparing likelihood and information.

The prior probability is the Wilson distribution of intensities, given in (3*a*) for the acentric case and in (3*b*) for the centric case (Wilson, 1949 ▸),
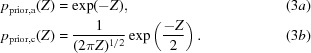



The probability distribution for the observed normalized intensity (*Z*_O_) given the true intensity is assumed to arise from Gaussian measurement error, with a standard deviation of 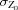. The probability distribution for observed intensities is then the convolution of the Wilson distribution with the Gaussian. This is given in (4*a*) and (4*b*) for the acentric and centric cases, reproduced from equations (9*a*) and (9*b*) from work on the LLGI intensity-based likelihood target (Read & McCoy, 2016 ▸),
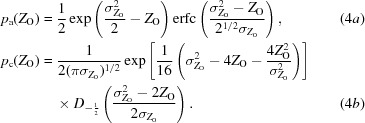



In these equations, erfc is the complement of the error function and *D* is a parabolic cylinder function (Whittaker & Watson, 1927 ▸).

For acentric intensities, the analytical solution to the information integral in (2) is given in (5), 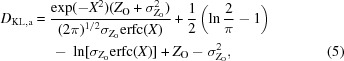
where 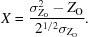



When the arguments give large positive values for *X*, both the exponential in the numerator of the first term and the complement of the error function in the denominator become extremely small, in which case it is preferable to use the scaled complement of the error function to obtain (6),
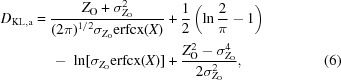
where erfcx(*x*) = exp(*x* ^2^)erfc(*x*).

There is also an analytical solution to the information integral for centric intensities, given in (7), 
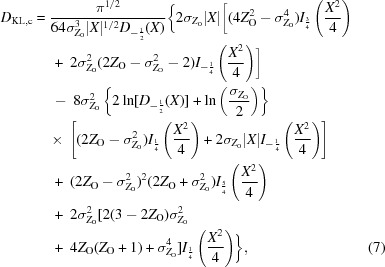
where 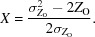



**Figure 1:**
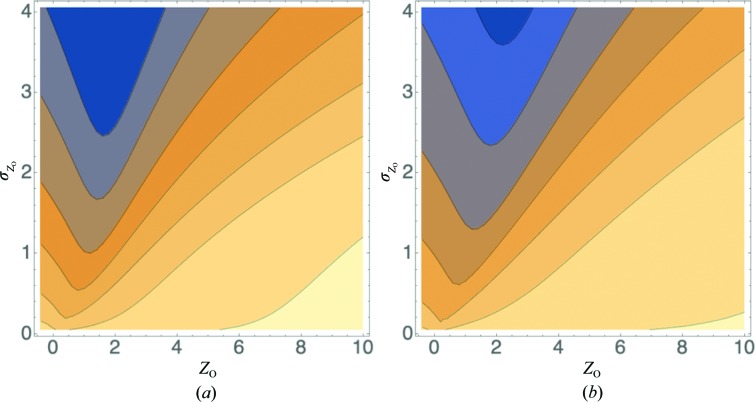
Contour plots showing bits of information in an intensity measurement as a function of *Z*_O_ and 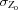 for (*a*) acentric and (*b*) centric intensity measurements. Contour lines are drawn, from the blue region through orange to yellow, at 0.01, 0.03, 0.1, 0.3, 1, 3 and 10 bits of information. This figure and Figs. 2–5 were prepared using *Mathematica* (Wolfram Research, Champaign, Illinois, USA).

However, it was judged easier to implement a numerical integral using functions that were already available in the computer code. To avoid numerical problems with overflow of the parabolic cylinder function for large negative arguments and underflow for large positive arguments, an exponential scaling is used where *Dx*_−1/2_(*x*) = exp[*x*(*x* ^2^)^1/2^/4]*D*_−1/2_(*x*). In addition, a change of variable from *Z* to *E* avoids a singularity at zero in the prior probability of the true intensity, giving (8), which can be evaluated using the expressions above,
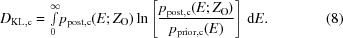



Fig. 1 ▸ illustrates the dependence of information gain on the normalized intensity and its standard deviation for both the acentric and centric cases. As one would expect, reflections with lower standard deviations convey more information. Reflections with higher intensity also have a lower prior probability and also therefore convey more information.

### 2.2. Implementation of information-gain calculation   

The calculation of information gain has been implemented within the program *Phaser* (McCoy *et al.*, 2007 ▸) and is available in versions from 2.8.2 by providing the keyword command ‘INFO ON’ in either the MR_AUTO or NCS modes. Our interest is in the information gained relative to the best estimate of the prior probability distribution of intensities, so the calculation is carried out after accounting for the statistical effects of both anisotropy and translational non­crystallographic symmetry, if present. The total number of bits of information conveyed by the data set is reported. In addition, the average number of bits of information per reflection is reported in resolution shells as a new indicator of the resolution dependence of data quality.

## 3. Relationship between KL-divergence and the log-likelihood gain score   

### 3.1. Information gain is equivalent to the expected log-likelihood gain score for a perfect model   

The expected log-likelihood gain, or eLLG, was originally defined as an integral over all possible pairs of observed and calculated structure-factor amplitudes consistent with the quality of the model and the standard deviation of each intensity measurement (McCoy *et al.*, 2017 ▸). This approach neglects the specific intensity values and so yields a very simple approximation that nonetheless allows valuable rules of thumb. For instance, the LLG that will be obtained for a partial model will be approximately proportional to the square of the model completeness, so that one can judge how much the signal will be reduced by searching separately for two domains. When defined in this way, the eLLG for a perfect model is equivalent to the expected information gain defined in our earlier work (Jamshidiha *et al.*, 2019 ▸), when that information is specified in units of nats.

Similarly, if the eLLG is expressed on a per-reflection basis that takes account of the actual measured intensity instead of averaging over all possible values, the actual information gain for a reflection (expressed in nats) corresponds to the eLLG for a perfect model. In other words, the information gained by a diffraction measurement defines an upper limit for the contribution that it could possibly make to the total LLG score. (9) defines an eLLG that averages over possible values of the calculated intensity weighted by their probability given the observed intensity, 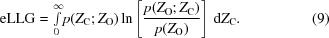



As above, Bayes’ theorem allows an alternative expression for the ratio in the argument of the logarithm, shown in (10), 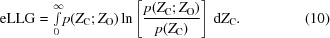



For a perfect model, the calculated structure factor is equal to the true structure factor, in which case (9) is equivalent to the KL-divergence in (2).

**Figure 2:**
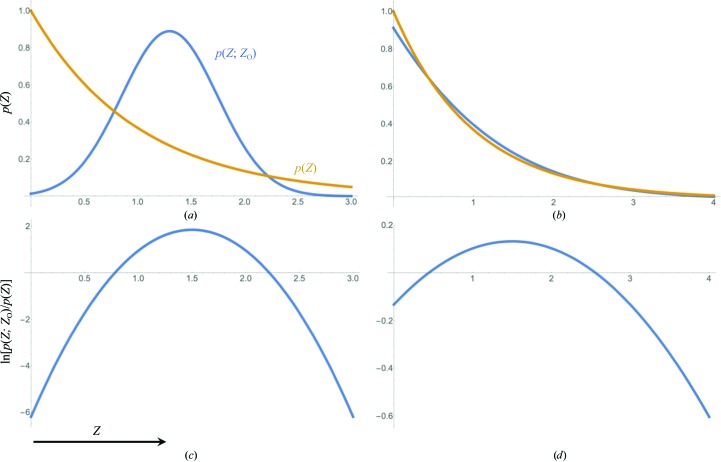
Illustration of information calculation for moderate and weak intensities. The top figures (*a*, *b*) illustrate the posterior probability of the true normalized intensity, *Z*, given the observed normalized intensity, *Z*_O_ (blue), as well as its prior probability before making the measurement (orange), while the bottom figures (*c*, *d*) show the corresponding logarithm of the ratio between the posterior and the prior probability. The information content is computed by integrating the log of the ratio (bottom figure) weighted by the posterior probability in blue above. The figures on the left (*a*, *c*) correspond to an intensity conveying 1 bit of information (*Z*_O_ = 1.5, 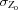 = 0.449), while the figures on the right (*b*, *d*) correspond to an intensity conveying 0.01 bits of information (*Z*_O_ = 1.5, 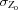 = 2.47).

Inspection of (2) and (10) shows that any value for the true structure factor that gives a positive contribution to the KL-divergence (or equivalently the eLLG for a perfect model) will also tend to be given a higher weight in the integral. This effect is illustrated in Fig. 2 ▸ for the cases of reflections with moderate and low information contents. When the information gain is low, no possible choice of the intensity calculated from even a perfect model will yield a high LLG score.

## 4. Correspondence between KL-divergence and *I*/σ ratios   

It might be useful to provide a very rough correspondence between the mean information gain in the highest resolution shell and the mean *I*/σ ratio. There is not, of course, a one-to-one relationship between these quantities. As seen in Fig. 1 ▸, in which observations with the same *I*/σ ratio will lie on a line running through the origin and observations with the same standard deviation will lie on a horizontal line, the information gain depends on both the intensity and its standard deviation. Nonetheless, we can obtain an intuitive idea of how these quantities are related by considering some drastic simplifying assumptions.

Firstly, we assume that the data do not suffer from significant anisotropy or tNCS, which will lead to dramatic variation in the *I*/σ ratios within the highest resolution shell. Secondly, we consider that near the resolution limit, the peak counts differ relatively little from background. In this case, the photon-counting statistics will be similar (and close to constant) in the peak and background regions, so that the standard deviations of the net integrated intensities (peak minus background) will be close to constant. Even then, we need a third assumption that different reflections in the shell have been measured with similar redundancy: averaging several measurements reduces the standard deviation of the mean by a factor equal to the square root of the number of measurements.

**Figure 3:**
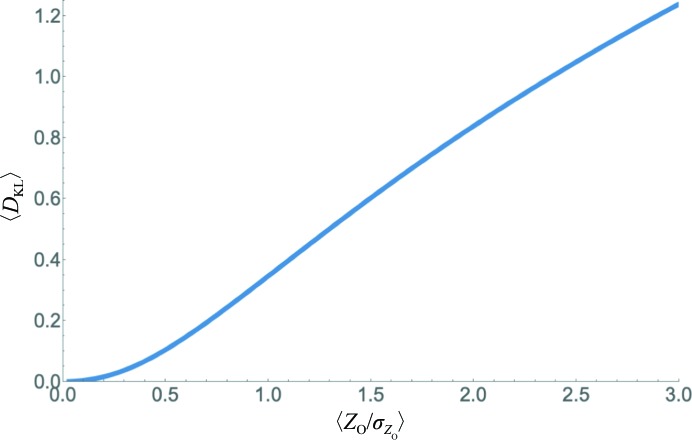
Approximate relationship between *I*/σ (represented by the ratio between the normalized intensity and its standard deviation) and the expected KL-divergence measured in bits per reflection. The calculation makes a variety of assumptions and should be taken only as a rough guide to the correspondence between these measures of signal to noise in data.

With these assumptions, we can compute the mean information gain expected for a shell of reflections with constant intensity standard deviation. The expected value is obtained by computing the KL-divergence (equations 5 and 6 for the acentric case) over all possible values of the observed normalized intensity, weighted by the probability of making that intensity observation (equation 4 *a* for the acentric case). This yields (11), which can be evaluated by numerical integration for a particular choice of the standard deviation of the normalized intensities, 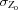. Note that if all of the intensity observations have the same standard deviation and the mean normalized intensity is 1, the mean *I*/σ ratio will be the inverse of 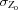, 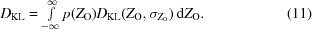



Fig. 3 ▸ shows the variation of this expected KL-divergence with the *I*/σ ratio. It can be seen that for very low *I*/σ ratios of less than about 0.2 the mean information gain will be close to zero, and will then increase steadily for higher ratios. To the extent that we can treat the curve in Fig. 3 ▸ as approximately linear, the mean KL-divergence will be very similar even if there is a limited distribution of intensity standard deviations over the observations in the shell, as long as these are uncorrelated with the intensity. An *I*/σ ratio of 1 corresponds roughly to a mean information gain of 0.35 bits, while an *I*/σ ratio of 2.5 corresponds to about 1 bit per observation. We have not carried out a systematic survey of data sets in the wwPDB (Berman *et al.*, 2007 ▸), but have noted that the mean information gain in the highest resolution shell is frequently in the range 0.5–1 bits, which is in agreement with these rough calculations because data are frequently cut with a threshold *I*/σ around 1 to 2.

## 5. Applications   

### 5.1. Pruning data by information gain   

In principle, if the effects of measurement error are accounted for properly in a well founded likelihood target, there should be no real disadvantage to including data with very little or even no signal, apart from wasting some computer time. In practice, most crystallographic methods still do not account optimally for measurement error, so it has been found that it can be helpful to limit the resolution to data containing significant signal (Karplus & Diederichs, 2012 ▸) or to prune data that are systematically weak because of effects such as anisotropy (Strong *et al.*, 2006 ▸). This can be understood by examining the effect of different treatments of measurement error. Model refinement, like molecular replacement in versions of *Phaser* prior to the introduction of the LLGI target (Read & McCoy, 2016 ▸), typically uses a likelihood target based on an inflated-variance Rice-function approximation (equation 12, acentric case) to add a contribution from measurement error to the contribution from model error (Murshudov *et al.*, 2011 ▸; Bricogne & Irwin, 1996 ▸), 
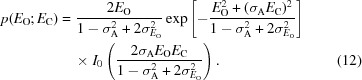



In this equation, *E*_O_ and 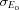 are the normalized observed amplitude and its standard deviation, obtained by some transformation from the observed intensity and its standard deviation. Most often, the algorithm of French & Wilson (1978 ▸) is used to compute the posterior value of the normalized amplitude and its standard deviation: *E*_FW_ and 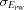. In the following, we will refer to the inflated-variance likelihood target based on (12) but using the French–Wilson amplitude estimates as LLG_FW_. We have shown that this approximation breaks down when measurement errors are large, whereas the LLGI target remains an excellent approximation to an exact likelihood target computed by numerical integration (Read & McCoy, 2016 ▸). This target is based on an alternative Rice-function approximation, shown for the acentric case in (13), 
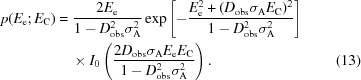



In this equation, *E*_e_ and *D*_obs_ are chosen to optimize the approximation by matching the first two moments of the exact distribution.

**Figure 4:**
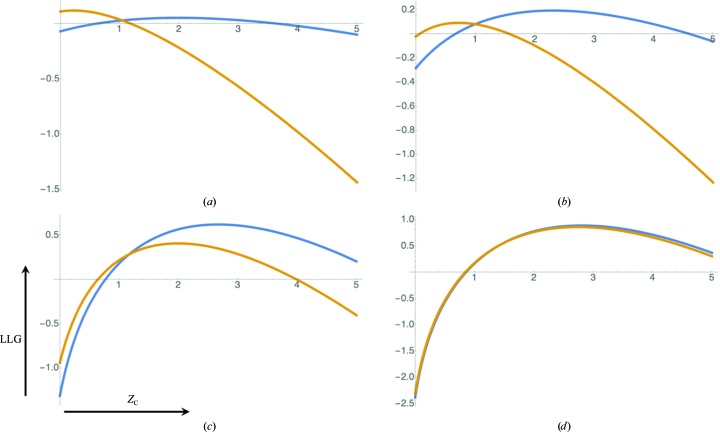
LLG as a function of the calculated normalized intensity, *Z*_C_, for several levels of information gain. In each case the observed normalized intensity, *Z*_O_, is 2.0 and the value of σ_A_ is 0.8, which would correspond, for instance, to an r.m.s. error of 0.4 Å for a model at a resolution of 2.2 Å. The LLG computed with the formula for LLGI (Read & McCoy, 2016 ▸) is shown in blue and the LLG computed with the inflated-variance Rice function LLG_FW_ is shown in orange. (*a*) Information gain is 0.01 bits, corresponding to 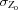 = 2.57, *E*_FW_ = 0.91, 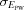 = 0.44. (*b*) Information gain is 0.1 bits, corresponding to 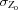 = 1.33, *E*_FW_ = 0.99, 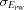 = 0.41. (*c*) Information gain is 1 bit, corresponding to 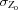 = 0.63, *E*_FW_ = 1.25, 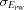 = 0.26. (*d*) Information gain is 3 bits, corresponding to 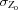 = 0.21, *E*_FW_ = 1.40, 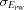 = 0.08.

Here, we show that the information gained by an intensity observation gives a good indication of whether or not the French–Wilson inflated-variance Rice target, LLG_FW_, will provide a sufficiently good approximation to the exact likelihood target. Fig. 4 ▸ compares the LLGI target with LLG_FW_ for observations with standard deviations corresponding to different information contents. As the information content drops even further than shown in this figure, the LLGI target becomes even flatter, yielding values very close to zero with very little dependence on the calculated structure factor, *i.e.* it ceases to influence any refinement or hypothesis test; on the other hand, LLG_FW_ continues to favour calculated amplitudes near the expected amplitude from the Wilson distribution. This justifies the omission of very weak observations from any calculation that uses LLG_FW_ or any other target that does not account well for the effect of measurement error.

Given that observations with very little information content have very little influence on likelihood calculations, such observations can legitimately be ignored to save computing time once the data have been characterized, even if the LLGI target is used. However, the fact that the systematically weak observations *are* weak provides information in characterizing anisotropy or tNCS, so they should be included in these steps of the analysis. This is the approach taken in *Phaser*, in which the anisotropy and tNCS analyses include all data, but data with low information gain are then excluded from subsequent calculations (Jamshidiha *et al.*, 2019 ▸). The current default is to exclude data conveying less than 0.01 bits of information according to the expected information criterion. Tests in the context of molecular replacement (where models are poor in challenging cases and such data would have even less influence than with good models) show that this is a good tradeoff. The use of the actual reflection-by-reflection information gain described here has also been tested, and gives comparable results at this information threshold (results not shown). When higher thresholds are applied, the reflection-by-reflection estimate is, as expected, more efficient in identifying the reflections that can be ignored with the least impact on the calculation.

#### 5.1.1. Effect of including weak data in the LLG_FW_ target   

The effect of using LLG_FW_ can be assessed by considering which likelihood scores will be encountered and how much they will differ from the scores that would be calculated with either the exact likelihood integral or the LLGI target. The largest errors will be encountered in the limiting case of a perfect model, which would have calculated structure factors corresponding to the true structure factors. Although we do not know the true intensities, we know their distribution of possible values consistent with an intensity measurement, *i.e.* the posterior probability distribution shown in (1). Knowing this, we can compute quantities such as the expected value of LLG_FW_ or, of greater relevance, the r.m.s. error in the LLG expected for the range of model structure factors that should be encountered.

For a particular intensity observation with a measured value and estimated standard deviation, we can compute an r.m.s. error with (14),
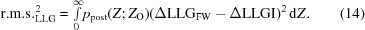



In this equation, the values represent the difference between the LLG value for a particular true intensity and the expected value over all possible true intensities given the observed intensity; this is used because reproducing the deviation from the mean is more important than reproducing the exact value when carrying out a search or testing a hypothesis.

**Figure 5:**
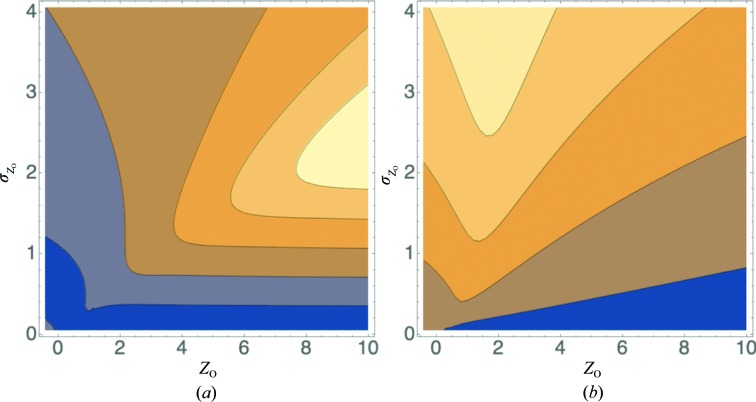
Contour plots illustrating, for the acentric case, the errors in likelihood scores for a perfect model arising from using French and Wilson amplitudes in the inflated-variance Rice likelihood target as a function of *Z*_O_ and 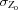. (*a*) The expected r.m.s. error in the likelihood score (converted to bits for comparison with information gain), averaged over the calculated structure factors consistent with the measurement, with contour lines drawn from the blue region through orange to yellow at 0.5, 1, 1.5, 2 and 2.5. (*b*) The ratio of the r.m.s. error from (*a*) and the information gain from Fig. 1 ▸(*a*), with contour lines drawn from the blue region through orange to yellow at 0.1, 1, 10 and 100. Only well measured intensities have likelihood errors that are smaller than the information content gained (points below the second contour line).

Fig. 5 ▸(*a*) shows the behaviour of this r.m.s. error as a function of both normalized intensity and standard deviation, expressed in terms of bits for easier comparison with the information measure. This confirms that LLG_FW_ is only a good approximation for relatively well measured data. Fig. 5 ▸(*b*) shows the relative error obtained by dividing the values in Fig. 5 ▸(*a*) by the information values in Fig. 1 ▸(*a*). This shows that the relative error becomes large when the information gain drops below about 1 bit.

Molecular-replacement calculations were carried out on a test case to evaluate the effect of these errors in practice. wwPDB (Berman *et al.*, 2007 ▸) entry 2g38 (Strong *et al.*, 2006 ▸) is the structure that inspired the development of the *UCLA Diffraction Anisotropy Server* (https://services.mbi.ucla.edu/anisoscale/), which can be used to prune weak data. The deposited data for this entry have already been pruned, but the complete data set was kindly provided by Mike Sawaya. These data were pruned at different information-content thresholds, and molecular-replacement calculations were carried out either with *Phaser* version 2.5.6 (the last release before the introduction of the LLGI target), using posterior amplitudes (French & Wilson, 1978 ▸) obtained from intensities with the *CCP*4 (Winn *et al.*, 2011 ▸) program *CTRUNCATE*, or with *Phaser* version 2.8.3, using intensity data. The structure contains two copies each of a 99-residue chain and a 198-residue chain. A search for a single copy of chain *A* of PDB entry 4w4k, 97% identical in sequence to the smaller chain (which comprises only 1/6 of the total structure), was carried out as a reasonably challenging problem.

The results are shown in Table 1 ▸. As expected, the signal increases for both targets when more data are added, as long as those data convey substantial useful information. A molecular-replacement solution is not found with either target when only data conveying 3 bits of information are used, but both succeed using data up to a 2 bit threshold. However, the LLG_FW_ target behaves more badly as weaker data conveying much less than 1 bit of information are added, increasing the noise level: the total score increases for both correct and incorrect solutions, with the correct solution eventually being lost once too many weak data have been added. On the other hand, the addition of weak data continues to improve the LLGI target even up to about a 0.01 bit threshold, and inclusion of even the weakest data does not jeopardize the solution. The results from this example suggest that if most deposited data have a mean information gain of 0.5 bits or more per reflection at their resolution limit (as discussed above), value could be gained from retaining even weaker data at high resolution, as long as the methods using these data account properly for the effects of measurement error.

### 5.2. Comparing approaches to pruning data   

A mode to analyse a diffraction data set and produce an output data file including the information measures will be made available in the new version of *Phaser* that is under development, *phasertng* (McCoy *et al.*, 2020 ▸). The original data will be left unaltered, on the principle that some programs (including *Phaser* itself) already make good use of unpruned data and that future algorithms may be able to extract even more from these data. Programs that have not been adapted to use the LLGI target could easily be changed to select data based on information thresholds; the relevant threshold may depend on the task at hand. Since the weak reflections provide the evidence for *which* reflections were weak (as opposed to being unobserved) in the original data, there is potentially a danger in discarding them and thereby hampering the refinement of anisotropy and tNCS parameters.

**Figure 6:**
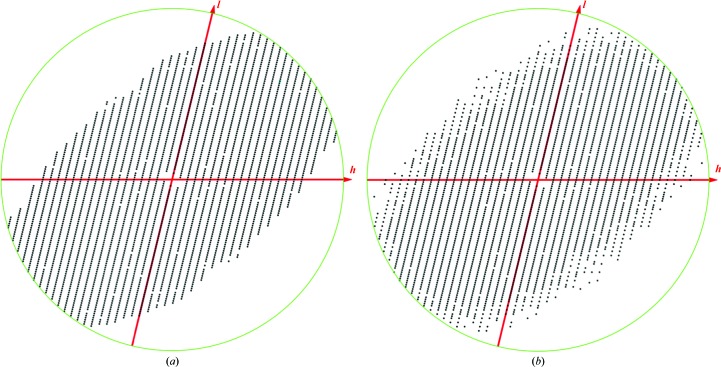
Result of pruning weak reflections from the human Rab27a data, illustrated for the *h*0*l* section. (*a*) The *STARANISO* server (Tickle *et al.*, 2018 ▸) retains 63 176 reflections from the full set of 91 204. (*b*) An information threshold of 0.2 bits retains 62 836 reflections. The boundary between the retained and pruned reflections is similar, but the information measure accounts for the systematic alternation of intensities arising from tNCS, keeping some strong observations that the *STARANISO* approach would delete and deleting some weak observations that would be kept. Figures were prepared with the *CCP*4 (Winn *et al.*, 2011 ▸) program *VIEWHKL*.

In other tools such as the *UCLA Diffraction Anisotropy Server* (Strong *et al.*, 2006 ▸) or *STARANISO* (Tickle *et al.*, 2018 ▸), pruning is carried out on the basis of smoothly varying functions such as overall anisotropy (*UCLA Diffraction Anisotropy Server*) or local signal to noise (*STARANISO*). In contrast, the information approach evaluates each intensity independently, taking account of the differing prior probability distributions for different reflections. When data are weak because of anisotropy, reflections with low information gain will still tend to be near to each other. In contrast, the effect of tNCS does not vary smoothly. Fig. 6 ▸ illustrates the difference that this makes for the human Rab27a data from PDB entry 6huf (Jamshidiha *et al.*, 2019 ▸).

## 6. Discussion   

As demonstrated here, including weak data in crystallographic calculations adds signal and can even make the difference between success and failure. With proper accounting for the effects of measurement errors, such as in the LLGI target used for molecular replacement in *Phaser*, even data with negligible signal can now be accommodated without the danger of adding noise. This allows structures to be determined more readily, even if they suffer from effects such as strong diffraction anisotropy or tNCS. The potential disadvantage of increasing computational cost without any added benefit can be avoided by using the close relationship between likelihood and information gain to identify the observations that can legitimately be ignored. However, when optimal treatments for measurement error are not used more care must be taken about which data to include.

It is important to account first for all systematic effects that might alter the distribution of the data, such as anisotropy and tNCS, because these are essential for defining the most accurate prior probability distribution. If information gain is calculated before correcting for these effects (implicitly assuming a radially symmetric distribution of expected intensities in reciprocal space), intensities that are systematically increased along the strong directions of diffraction or enhanced by constructive interference from tNCS will appear to convey more information. On a related note, if the estimated standard deviations are underestimated, observations will also appear to convey more information; this is more likely to be an issue for serial crystallography, where data processing is less mature than for single-crystal diffraction.

The methods described here could in principle be improved further by accounting for other effects that change the intensity distributions, such as lattice-translocation defects or twinning. Twinning, in particular, reduces the variance in the intensity distribution, which should be accounted for in both the prior and posterior probability distributions. Such a treatment would quantify our understanding of how the presence of twinning reduces the information available from a data set.

Finally, we are deeply concerned about the trend for crystallographers to deposit data that have been pruned, corrected for anisotropy and even sharpened to bring the diffracting power in the weaker directions up to that in the strongest direction. While such treatments can improve the subjective interpretability of maps, they could be problematic for any methods using statistically based scoring functions. For instance, an isotropic *B* factor that might have been positive when refined against unaltered data will potentially become negative when refined against sharpened data.

## Figures and Tables

**Table 1:** Effect of information-content thresholds on maximum-likelihood molecular-replacement calculations using the LLG_FW_ and LLGI targets

		LLG_FW_			LLGI		
Information threshold (bits)	No. of reflections	Top correct	Top incorrect	Ratio	Top correct	Top incorrect	Ratio
3	12346	†	73.7	0	†	72.7	0
2	15765	119.7	81.0	1.48	123.5	79.8	1.55
1	19771	131.0	83.2	1.57	130.4	82.6	1.58
0.5	22278	142.5	81.9	1.74	140.0	76.9	1.82
0.1	25186	169.9	97.7	1.74	151.5	72.6	2.09
0.01	27516	222.3	164.3	1.35	152.9	74.1	2.06
0.001	29133	272.9	258.0	1.06	152.9	73.4	2.08
0	32631	†	439.3	0	152.1	74.0	2.06

† The correct solution was not found in this molecular-replacement search.
